# Amplified Drought and Seasonal Cycle Modulate *Quercus pubescens* Leaf Metabolome

**DOI:** 10.3390/metabo12040307

**Published:** 2022-03-30

**Authors:** Amélie Saunier, Stéphane Greff, James D. Blande, Caroline Lecareux, Virginie Baldy, Catherine Fernandez, Elena Ormeño

**Affiliations:** 1 Aix Marseille Univ., CNRS, IRD, Avignon Univ., IMBE, 13331 Marseille, France; stephane.greff@imbe.fr (S.G.); caroline.lecareux@imbe.fr (C.L.); virginie.baldy@imbe.fr (V.B.); catherine.fernandez@imbe.fr (C.F.); elena.ormeno-lafuente@imbe.fr (E.O.); 2 Department of Environmental and Biological Sciences, University of Eastern Finland, P.O. Box 1627, 70211 Kuopio, Finland; james.blande@uef.fi

**Keywords:** amplified drought, Mediterranean forest, metabolome, flavonoids

## Abstract

The intensification of summer drought expected with climate change can induce metabolism modifications in plants to face such constraints. In this experiment, we used both a targeted approach focused on flavonoids, as well as an untargeted approach, to study a broader fraction of the leaf metabolome of *Quercus pubescens* exposed to amplified drought. A forest site equipped with a rainfall exclusion device allowed reduction of natural rainfall by ~30% over the tree canopy. Leaves of natural drought (ND) and amplified drought (AD) plots were collected over three seasonal cycles (spring, summer, and autumn) in 2013 (the second year of rain exclusion), 2014, and 2015. As expected, *Q. pubescens* metabolome followed a seasonal course. In the summer of 2015, the leaf metabolome presented a shifted and early autumnal pattern because of harsher conditions during this year. Despite low metabolic modification at the global scale, our results demonstrated that 75% of *Quercus* metabolites were upregulated in springs when trees were exposed to AD, whereas 60 to 73% of metabolites (93% in summer 2015), such as kaempferols and quercetins, were downregulated in summers/autumns. Juglanin, a kaempferol pentoside, as well as rhododendrin derivatives, were upregulated throughout the year, suggesting an antioxidant ability of these metabolites. Those changes in terms of phenology and leaf chemistry could, in the end, affect the ecosystem functioning.

## 1. Introduction

By the end of the 21st century, climatic models applied in the Mediterranean region predicted a rise in temperature from 2 to 6 °C, depending on the climate scenario and the season; a decrease in annual rainfall of ~30% especially in summer; and an increase of summer drought duration [1,2,3]. This region is therefore one of the most sensitive to climate change in terms of warming and drying. Plants may modify their metabolism to counteract the climate-related oxidative stress [4], especially when drought becomes recurrent over a period of years [5,6]. 

Numerous studies have shown that secondary metabolites, such as terpenoids [7,8,9] or phenolic compounds [10,11], are involved in drought resistance. Primary antioxidants (e.g., glutathione and tocochromanol [4]) and amino acids, as well as osmoprotectants (e.g., prolie and ectoine [12,13]), carbohydrates [14,15], and signal molecules (e.g., abscissic acid [16,17]), can also shift under water deficit [18]. Evaluating a plant’s capacity to resist water deficit at the organism level requires an integration of a large part of the plant’s metabolome [19]. Targeted metabolic profiling provides information on plant resistance to water stress. Phenolic compounds (e.g., simple phenols such as phenolic acids or polyphenols such as flavonoids and tannins) are thus known to play a key role in stress tolerance related to their antioxidant properties [20,21]. Such approaches are nevertheless limited to the study of restricted and identified foliar phenolics for quantitative purposes during short periods of water deficit [10,11], whereas studies integrating metabolite regulations over long periods of water deficit are still limited. Untargeted metabolomics approaches are required to evaluate the global impact of stresses on plant metabolite production. To date, these approaches have been used to assess how plants respond to short periods of heat stress [22,23], oxidative stress [24], high salinity [25,26], and high radiation [27,28]. Numerous studies have also applied untargeted approaches to assess the metabolome changes in response to drought stress, especially in laboratory experiments [29,30,31] and, to a lesser extent, over short-term periods in the field [32,33]. Long-term studies on the effects of drought are still scarce and based on a limited number of plant models [34]. There are several untargeted techniques for conducting metabolic analyses (e.g., GC-MS, LC-MS, and NMR). However, the large variety of plant metabolites detected with these methods, together with the difficulty in identifying and quantifying them, induces many uncertainties [35] and explains the lack of knowledge about the global response of plant metabolomes to recurrent water deficit. 

Our previous studies demonstrated that amplified drought (AD) induced little change in the concentration of ubiquitous primary antioxidant metabolites of *Quercus pubescens* Willd. when subjected to AD in the field after 2 to 4 years of application, despite the remarkable decline of leaf water potential, net photosynthesis, and stomatal conductance [36,37]. In the present study, we focused our efforts on evaluating whether the metabolome of *Q. pubescens*, mainly its secondary metabolism, is modified under AD compared to natural drought (ND) over the same period. The downy oak leaf metabolome was studied using two approaches: a targeted analysis focused on leaf phenolic compounds (mainly flavonoids) for a first glance at the metabolome modification, and an untargeted metabolomics approach for a broad overview of metabolic changes. It is hypothesized that stress related to AD induces metabolic changes in *Q. pubescens* at the leaf level that can be assessed through targeted and untargeted metabolomics approaches. 

## 2. Results

### 2.1. Ecophysiological Parameters

Results on gas exchanges and water potentials were fully described by Saunier et al. [36]. In summary, amplified drought clearly limited tree gas exchange, especially in 2015 due to the harsher climatic conditions. These results were linked to the reduction of water availability in soil, and not to a vapor-pressure deficit (VPD), which remained unchanged among treatments (Table S1). The carbon (C) and nitrogen (N) contents of leaves also remained stable, except for nitrogen levels that decreased in autumn 2015 without any effect on the C:N ratio.

### 2.2. Leaf Metabolic Profiles of Phenolic Compounds

**Figure 1:**
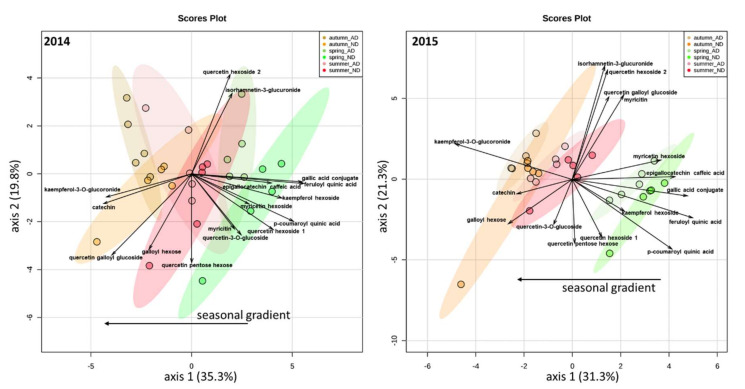
Principal component analysis (PCA) on phenolic compounds (targeted analyses) according to years and seasons in 2014 (**left**) and 2015 (**right**).

A targeted approach was first performed to specifically measure the effects of AD on some phenolic compounds, such as flavonoids. The metabolic profiles were mainly influenced by seasonality (*F* = 7.7 and 7.2 in 2014 and 2015, respectively, *p* < 0.001, PCA, and PERMANOVA, Figure 1 and Table 1) and, to a lesser extent by drought (*F* = 3.9 and 2.8 in 2014 and 2015, respectively, *p* < 0.01 and *p* < 0.05). No differences were detected comparing the metabolic profiles of leaves sampled in ND vs. AD plots, whatever the season or year considered, except in the spring of 2015 (see the pairwise comparisons season-by-season in Table S2 in the Supplementary Materials). 

Based on the low effect of drought treatments at the global scale, an univariate analysis was conducted to reveal any significant changes in individual compound concentrations relative to water deficit. All phenolic compounds analyzed through the targeted analysis, with their concentrations, are presented in the Supplementary Materials (Table S3). Several phenolic concentrations were decreased by drought (Student’s tests, *p* < 0.05), such as myricitrin for which concentrations decreased by 32% and 26% in the spring of 2014 and 2015, and by 58% and 36% in the summer of the same years, respectively. Many quercetin derivatives also presented lower concentrations under drought, especially in 2014, such as quercetin-3-*O*-glucose in the spring of 2014 (−36%), a quercetin pentose hexose in the summer of 2014 (−50%), quercetin galloyl glucose in the autumn of 2014 (−45%), and quercetin hexose 1 in the spring of 2015 (−33%). Feruoyl quinic acid decreased by 32% and 25% in the autumn of 2014 and 2015, respectively, whereas *p*-coumaroyl quinic acid decreased by 34% in the spring of 2014. 

Only one compound annotated as a kaempferol hexose presented higher concentrations with drought in the summer of 2015 (+50%).

### 2.3. Leaf Metabolic Fingerprints

#### 2.3.1. Detected Specialized Metabolites

A metabolic network, based on compound exact mass and MS^2^ spectra similarities, was constructed to annotate the major compounds detected by the untargeted approach (Figure S1). Detected metabolites generally belonged to phenylpropanoid pathway, such as cinnamic acid derivatives (e.g., chlorogenic acid and caffeoyl hexoses), water soluble tannins (e.g., mono-, di-, and trigalloyl hexoses), flavonol glycosides (e.g., mainly quercetin and kaempferol derivatives), and flavanols (e.g., epicatechin and epigallocatechin). 

#### 2.3.2. Metabolic Shifts According to Drought and Seasons

**Figure 2:**
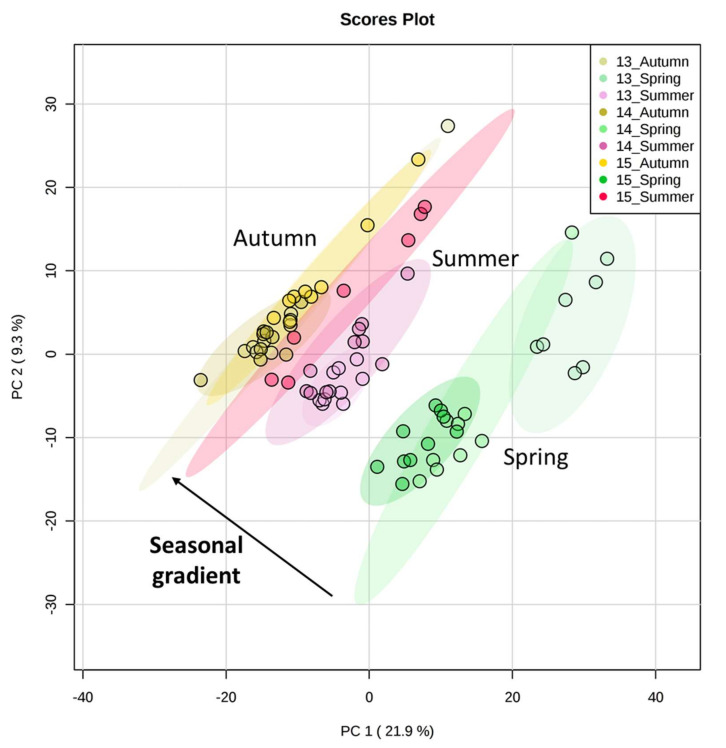
Principal component analyses (PCA) on metabolic fingerprints (untargeted analyses) according to years and seasons. The drought treatments were merged for this analysis.

As for the targeted analysis, *Q. pubescens* metabolic fingerprints did not show any significant variation under AD compared to ND for all years (*F* = 1.3, 1.5 and 1.2 in 2013, 2014, and 2015 respectively, *p* > 0.05, Table 1, Figure 2) with a higher variation due to seasons (*F* = 7.0, 4.7 and 3.9 in 2013, 2014, and 2015 respectively, *p* < 0.001). During 2015, when harsher conditions were recorded, the fingerprints of the summer-sampled leaves (ND and AD plots) shifted to an autumnal pattern, with a similar metabolic composition of autumn-sampled leaves (Table S4, Figure S2).

#### 2.3.3. Downregulated and Upregulated Drought Metabolites

**Figure 3:**
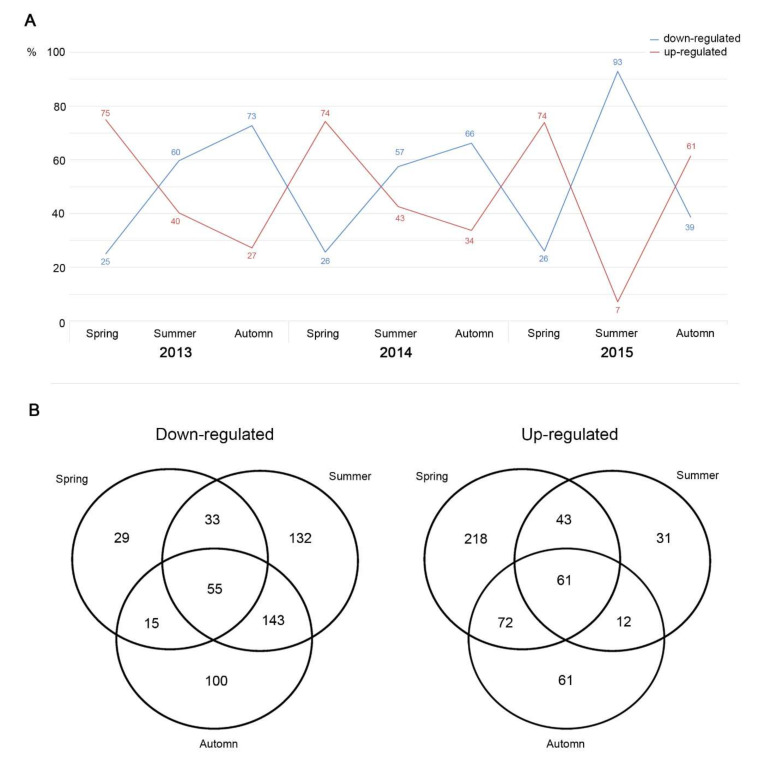
(**A**) Percentage of upregulated (in red) and downregulated (in blue) features according to years and seasons. (**B**) Venn diagram giving the repartition of downregulated (**left**) and upregulated (**right**) metabolites under drought according to seasons (with merged years).

The construction of heatmaps for each season/year permitted the listing of downregulated and upregulated features relative to drought (VIP score > 1, supplementary excel file—metabolites annotation). As demonstrated for the descriptive analysis realized above, the feature levels of expression were strongly dependent on seasons (Figure 3A). Briefly, AD induced an upregulation of 75% of the detected features in spring relative to ND, with 25% of them being downregulated. In summer, 57% to 60% of the features were downregulated, with an increase in this proportion in autumn (66% to 73% of the whole set of detected features) and with an opposite course of the upregulated features. Again, the level of downregulation of the metabolome was quite exceptional in the summer of 2015, with 93% of downregulated features in AD plots. 

Venn diagrams were then used to determine whether dysregulations of the features were season-specific or ubiquitous throughout the year (Figure 3B), providing eight datasets at the end of the process. Features were tentatively annotated though raw formula determinations, with searches in non-spectral databases, and an experimental MS^2^ spectrum (when available) was compared with in silico generated spectra and/or an online spectrum database (see the Supplementary Materials, “Supplementary excel file: metabolites annotation”).

Some representatives of downregulated and upregulated metabolites were grouped in Table 2 and Table 3, respectively. Among downregulated metabolites, quercetin and kaempferol derivatives were frequently detected, especially in summer and autumn. Interestingly, some metabolites, such as kaempferol hexoside (M448T758, likely astragalin) may exhibit opposite dysregulation according to seasons. 

Regarding upregulated metabolites, many rhododendrin derivatives (apiosyl rhododendrin, epirhododendrin, rhododendrin gallate, and rhododendrin, Figures S3–S5) were underscored whenever the leaves were sampled. In the same way, kaempferol pentoside (M417T860, likely juglanin) was unequivocally upregulated in all seasons and years with amplified drought.

## 3. Discussion

Amplified drought leads to a decrease of carbon assimilation, stomatal closure, and transpiration under AD, as discussed in Saunier et al. [36], with a marked decline of gas exchanges in 2015 when harsher climatic conditions were recorded compared to 2014. The study of Saunier et al. [36], performed concomitantly with the present study, showed that such gas exchange limitations under AD were associated with a reduction of water availability in soil (indicated by a decrease of predawn water potential), and were not linked to vapor-ressure deficit (VPD), which remained unchanged among treatments. *Quercus pubescens* is well known to have a high stem efficiency, allowing for the maintenance of net photosynthesis under drought stress [38]; however, it seemed that, with harsher climatic conditions (in 2015), the limits of this capacity were reached [36]. Despite trees being submitted to a marked AD, the leaf C content and N content, and the C:N ratio, remained rather stable between AD and ND. These results suggest that nitrogen assimilation was not affected by recurrent drought; even after 3 to 4 years of application, the levels of leaf nitrogen concentrations observed in our study were in agreement with previous studies [39,40]. Thus, the metabolite dysregulations observed in this study were most likely not linked to nitrogen assimilation, since several studies have shown that a high level of nitrogen may lead to a substantial reduction in leaf phenolic concentrations [41,42]. 

Both targeted and untargeted analyses suggested a slight-to-no effect of amplified drought on *Quercus pubescens* metabolomes as a whole. Leaf metabolomes shifted towards an autumnal composition in the summer of 2015, both for natural and amplified droughts, suggesting a premature metabolic slow-down when the harshest conditions were reached. This shift could imply some effects in plant and ecosystem functioning, such as an acceleration of plant senescence. This phenomenon is well known under drought and contributes to a nutrient remobilization from old leaves to the rest of the plant [43]. 

Taken season by season, trade-offs could affect some specific biosynthetic pathway, as upregulation of metabolites was marked in spring, whereas downregulation occurred in summer and autumn under drought application. Some kaempferol hexosides could be downregulated, especially in summer and autumn, whereas a kaempferol pentoside, likely juglanin, already reported as an antioxidant [44], was upregulated throughout the year, suggesting balanced biosynthetic pathways and/or flavonoid remobilization to increase leaf antioxidant capabilities. This difference of response of *Q. pubescens* facing drought seems to be dependent on resource availabilities, and may also be linked to phenological processes (i.e., the development of new leaves in spring [34,45]). This trade-off could be also linked to the ability to scavenge reactive oxygen species (e.g., ROS, H_2_O_2_), especially in a context of reduced carbon availability. We could say that this kaempferol derivative was more efficient in terms of scavenging under summer and autumn conditions. Amić et al. [46] showed a higher experimental radical scavenging activity for kaempferol and its glycosides (monohydroxylated—one OH group on the B ring) than quercetin and its glycosides; dehydroxylated—two OH groups on the B ring). However, the antioxidant role of kaempferol compared to that of quercetin remains unclear, since Ryan et al. [47] and Tattini et al. [48] demonstrated that quercetin has a better ability to scavenge ROS produced under drought stress, compared to kaempferol. Those discrepancies on antioxidant activity can also be explained by the different types of assays that can be performed to evaluate the scavenger activity of phenolic compounds. We can tell that such trade-offs also occurred between other phenolic compounds, according to their efficiency in acting as ROS scavengers and the stress conditions.

Even if seasonality triggered the metabolic response under drought, some features, such as those of rhododendrin and apiosyl rhododendrin, seemed to play a key role in the defense system of *Q. pubescens* with an upregulation throughout the year. Therefore, those features could be considered as potential drought biomarkers. The role of rhododendrin derivatives (which are phenolic glycosides) as defense compounds in plants was only hypothesized by Santamour and Lundgren [49] in cases of herbivore attacks in *Betula* spp. Those compounds could also act as defense compounds against abiotic stresses. It has been demonstrated that some compounds can be involved in responses to both biotic and abiotic stresses [50]. The systematical downregulation of some features (e.g., quercetin apiosyl hexose, kaempferol glucuronide, and, to a lesser extent, myricitrin) over seasons suggests that their antioxidant ability was lower than that of rhododendrin derivatives under drought. Moreover, this potential trade-off could have been enhanced by the carbon limitation highlighted through the decrease of net photosynthesis [36]. Downregulation could also be explained by the oxidation of those phenolic compounds through catabolic-related reactions, as has been demonstrated by Hernández et al. [11] on *Camellia sinensis*. In the latter study, an increase in quinone concentrations was the result of epicatechin and epigallocatechin oxidations, which are able to scavenge ROS. That could indicate an increase of antioxidant activity from epicatechin and epigallocatechin. Thus, in our case, the decreases of quercetin apiosyl hexose, kaempferol glucuronide, and myricitrin could indicate a better scavenging capacity of ROS and those compounds could then be also considered as drought biomarkers. In order to verify this hypothesis, further investigations are required to look for oxidation products in our samples. 

Our results differ from those of Rivas-Ubach et al. [34], where they mainly highlighted an upregulation of phenolic compounds under drought on *Q. ilex* leaves, another Mediterranean species. These differences could be explained by the primary antioxidant system of *Q. pubescens*, as shown in our previous study [36], which showed a strong accumulation of tocochromanols, with maximum concentrations in autumn. Those compounds belong to the universal antioxidant system in plants and are involved in leaf protection against oxidative damage [51]. Since tocochromanols and phenolic compounds share substrates [52], it is possible that those two biosynthesis pathways compete with each other. If so, the maintenance of tocochromanols contents could avoid a too-important investment in phenolic compounds, which could explain the slight changes observed in our study. Moreover, trees sampled by Rivas-Ubach et al. [34] were stressed for a longer time than they were in our study (10 years), which can also explain our different results. It has been demonstrated that *Q. ilex* growth was only affected after 7 years of recurrent drought [53]. Non-perturbed ecosystems require long-term periods of climate change (7–14 years) to significantly endanger their functioning or to cause loss of their resilience capacity [54]. Ongoing research with rain restriction over longer time periods is thereby necessary to evaluate whether climate change will impact *Q. pubescens* forests, since the duration of drought stress could modulate *Q. pubescens* metabolome. 

In addition, our study mainly focused on phenolic compounds, but it could be interesting to look deeper into primary metabolism to better understand the metabolic changes of *Q. pubescens* and the trade-offs under recurrent drought. Gargallo-Garriga et al. [32] showed that water deficit, applied in a natural area, strongly affected the leaves of *Alopecurus pratensis* and *Holtus lanatus*, two grassland species, resulting in increased concentrations of osmosprotectant compounds (e.g., sugars). Urano et al. [55] showed high accumulations of amino acids in the leaves of *Arabidopsis thaliana* under lethal drought stress conditions. Even under mild drought stress, the *A. thaliana* metabolome showed an increase in proline as well as a decrease in aspartate [56]. A similar accumulation in terms of carbohydrates was also demonstrated in *Q. ilex* under drought [34].

## 4. Materials and Methods

### 4.1. Experimental Site

This study was conducted at the Oak Observatory at the Observatoire de Haute Provence (O_3_HP), which was created in 2009 in order to study the *Q. pubescens* forest ecosystem. The experimental site, located 60 km north of Marseille (5°42’44’’ E, 43°55’54’’ N), as well as the rain exclusion system, is fully described by Saunier et al. [36]. In this site, natural rain has been reduced by ~30% since April 2012, in an attempt to mimic climatic model projections for the end of this century [1,2,3]. Rainfall exclusion data, available in the study by Saunier et al. [36], show that the drought period was extended by 2–4 months and that the 4th year (2015) of the experiment was drier than the 3rd year (2014).

Five trees were surveyed per plot during each seasonal campaign in 2014 and 2015 for the targeted analyses (corresponding to the 3rd and 4th years, respectively, after the beginning of rain exclusion). The untargeted analyses were performed on the same leaves during the 2nd year of rain exclusion (the spring, summer, and autumn of 2013). Leaves were collected from fully sun-exposed branches at the top of the canopy. 

Photosynthetically active radiation (PAR, µmol m^−^^2^ s^−1^) and temperature (°C) were recorded every minute and every 2 min, respectively, one week before, and during, the field campaigns during 2014 and 2015 (Table 4). Data were collected with a quantum sensor (PAR-SA 190^®^, LI-COR, Lincoln, NE, USA) and a temperature probe (CS215, Campbell Scientific, North Logan, UT, USA) was placed at the top of the canopy. The values presented in this study were averaged between 12:00 and 15:00 (local time) for the whole period considered. During our field campaigns, the PAR was similar in the spring and summer for both years (around 1200–1250 and 1350–1380 µmol m^−^^2^ s^−1^, respectively). In contrast, the autumn of 2014 was cloudier than the autumn of 2015, leading to lower PAR values (675 and 1015 µmol m^−^^2^ s^−1^, respectively). In terms of temperature, the spring and summer of 2015 were warmer compared to 2014 (+2.5 °C in spring and +3.3 °C in summer), whereas the mean temperatures in the autumn of 2014 and 2015 were similar (19.4 and 19.9 °C, respectively).

### 4.2. Leaf Sampling

During each field campaign, ten leaves were collected per tree. Prior to any extraction, the leaves of each tree were pooled together, frozen in liquid nitrogen, stored at −80 °C in the laboratory, and finally freeze-dried and ground into powder (MM 400, Retsch, Haan, Germany). 

### 4.3. Carbon and Nitrogen Leaf Concentrations

Organic carbon (C) and total nitrogen (N) contents were determined after the thermal combustion of leaf powder (5 mg DM) on a Flash EA 1112 series C/N elemental analyzer (ThermoScientific, Waltham, MA, USA) [57].

### 4.4. Leaf Metabolic Profiles of Phenolic Compounds (Targeted Metabolomics)

Ten mg (DM) of each ground leaf sample was mixed with 1 mL of methanol containing 1% formic acid. The extract was homogenized for 5 min in an ultrasonic bath and centrifuged at 12,000 tr min^−1^ for 5 min. Analyses were performed with a UHPLC-TQD (an Acquity UPLC autosampler and a thermostated column compartment and UV diode array, Waters, Milford, MA, USA). UHPLC separation occurred on a C18 BEH column (2.1 mm × 150 mm, 1.7 µm, Waters, USA). The elution rate was set to 0.4 mL min^−1^ at a constant temperature of 30 °C. Injection was set to 2 µL. Chromatographic solvents were composed of (a) water with 0.1% formic acid, and (b) acetonitrile with the same eluent additive. The chromatographic gradient was defined as follows: 3% of B for 3 min, then increasing the solvent B proportion to reach 90% at 17 min. Each analysis was followed by a phase of column cleaning at 90% B for 3 min and column equilibration for 6 min, providing a total runtime of 25 min. The photodiode array was set from 190 to 600 nm and flavonols were detected at 350 nm. Their identity or structure was confirmed with the triple quadrupole mass detector in full scan negative ionization mode only, since only a few compounds were detected in positive ionization mode. The capillary voltage was 2.9 kV, the cone voltage was 35 V, the source temperature was maintained at 150 °C, and the desolvation temperature was maintained at 400 °C. An external quantification with monoglycosylated flavonols (quercetin and myricitrin) and hydroxycinnamic acid standards was applied. In the end, our data set included 16 compounds.

### 4.5. Leaf Metabolic Fingerprints (Untargeted Metabolomics)

#### 4.5.1. Metabolite Extraction

Two hundred mg (DM) of each sample was suspended in 4 mL of methanol:water (50:50) and subjected to ultrasonication for 5 min at room temperature. Extracts were then filtered using a syringe filter (PTFE 13 mm, 0.22 µm, Restek, Bellefonte, PA, USA). Analyses were performed with an UHPLC instrument (Dionex Ultimate 3000 equipped with an RS Pump, an autosampler, a thermostated column compartment, and a UV diode array, Thermo Scientific, Waltham, MA, USA) coupled to an accurate mass spectrometer (qToF) equipped with an ESI source (Impact II, Bruker Daltonics, Billerica, MA, USA). UHPLC separation was carried out on an Acclaim C18 column (150 mm × 2.1 mm, 2.2 µm, Thermo Scientific, MA, USA). The elution rate was set to 0.5 mL min^−1^ at a constant temperature of 45 °C. A pooled sample combining 10 μL of each sample was used to determine the chromatographic method and the injection volume. This pooled sample was also used as a quality control. Injection was set to 10 µL after a dilution by two of all the extracts with the same solvent as the one used for extraction. Chromatographic solvents were composed of (a) water with 10 mM of ammonium formate, and (b) acetonitrile/water (95:5) with the same eluent additive. The chromatographic gradient was defined as follows: 5% of B for 2 min,then increasing the solvent B proportion to reach 20% at 10 min, maintained during 4 min. Each analysis was followed by a phase of column cleaning at 100% B for 3 min and column equilibration for 3 min, providing a total runtime of 20 min. Samples of each condition were randomly injected to integrate time-dependent MS fouling. Pooled samples, injected at the beginning, at the end, and for every 6 samples, were used for ion intensity normalization. Blanks were also injected to remove features linked to background. MS parameters were set as follows: nebulizer gas, N_2_ at 51 psi; dry gas, N_2_ at 12 L min^−1^; capillary temperature at 200 °C; and voltage at 3000 V. The mass spectrometer was systematically calibrated with a formate/acetate solution forming clusters on the studied mass range before a full set of analysis. The same calibration solution was automatically injected before each sample for internal mass calibration. Because the negative mode provided better sensitivity, mass spectra were recorded in this ionization mode, in full scan mode, from 50 to 1200 amu at 2 Hz. DDA-MS^2^ analyses were performed on the three major features detected at each scan, and on a pooled sample for metabolite annotation. 

#### 4.5.2. Data Analyses

Analyses were automatically recalibrated using internal calibration, ensuring a precision of *m*/*z* lower than 2 ppm on the mass range, before exporting data in netCDF files (centroid mode) using Bruker Compass DataAnalysis 4.3. Analysis files were then processed using the XCMS package [58] of R software, using the different steps necessary to generate the final data matrix: (1) peak picking for detection of different features; (2) retention time correction (method = “obiwarp”); (3) grouping; (4) filling peaks to integrate portions where peaks were initially absent; and (5) reporting and transferring the data matrix generation to Excel. Each individual ion of each analysis was then normalized according to the injection order, as described by VanDerKloet et al. [59]. After the data set normalization, around 6000 features were kept before the filtering steps. Then, to ensure data quality and to remove redundant signals, three successive filtering steps were applied to preprocessed data using an in-house script on R. The first filtering step was based on the signal/noise (S/N) ratio, in order to remove features observed in blanks (S/N set at 10 comparing the pooled samples and blanks). The second filtering step allowed suppression of features that presented a variable intensity in pooled samples (threshold at 0.3). The last step consisted of the deletion of all auto-correlated features (threshold at 0.8) to remove isotopes and adducts. In the end, 799 ions were kept for data analyses. Molecular networks based on MS^2^ spectra from 2013–2014 (reference years according to drought) were constructed with GNPS [60] and observed under Cytoscape 3.7.1 [61] in order to highlight the major detected metabolites and their seasonality.

#### 4.5.3. Drought Biomarker Annotation

Features were tentatively annotated though raw formula determination with searches in non-spectral databases (SciFinder) and an experimental MS^2^ spectrum (when available), compared with in silico-generated spectra (MetFrag) and/or an online spectrum database (Metlin). Mass spectra of drought biomarkers recorded after untargeted analysis were specifically acquired in both negative and positive mode to tentatively annotate them. Based on the recorded mass spectra, rhododendrin (Ambinter c/o Greenpharma, Orleans, France) was co-injected to confirm its identification. 

### 4.6. Statistical Analyses

All the multivariate and univariate analyses were performed with R software (version 3.5.2) and multivariate analyses were drawn under metaboanalyst [62]. Principal component analyses (PCAs, package ade4 on log-transformed and auto-scaled data) were performed, targeted and untargeted, to distinguish the different treatments according to years and seasons. Then, PERMANOVA testing (package vegan on log-transformed and auto-scaled data) was performed on the two data sets (targeted and untargeted metabolome datasets) taking into account all metabolites (or detected features) for each year to highlight the effect of seasons and treatments, with 999 permutations. Pairwise tests (packages RVAideMemoire and pls [63]) were performed to test differences between groups according to treatments and seasons for each year (999 permutations). Student’s tests according to the year and the seasons were performed between treatments to determine the effect of drought on phenolic compounds analyzed in the targeted analyses.

To highlight the regulation of *Quercus pubescens* metabolome under drought, heatmaps were built according to treatments for each season and each merged year with features showing a VIP score above 1. Then, results obtained for AD were extracted and used to build Venn diagrams through Venny (https://bioinfogp.cnb.csic.es/tools/venny/; 21 September 2021) according to seasons for upregulated and downregulated metabolome. 

## 5. Conclusions

*Quercus pubescens* metabolome was mainly affected by seasons with a shift towards similar metabolite profiles in summer and autumn under harsher and repeated drought conditions. Even the *Q. pubescens* response to drought was highly dependent on seasons, with a different upregulation or downregulation according to the seasons (mainly between summer and autumn). Those results suggested that seasonality plays a key role in phenolic compounds trade-offs, probably due to their different abilities in scavenging ROS (i.e., the difference between kaempferol and quercetin).

Our study also highlighted that some compounds were upregulated (rhododendrin, apiosyl rhododendrin) and downregulated (quercetin apiosyl hexose, kaempferol) under drought, regardless of the season; therefore, they could be considered as drought biomarkers. Overall, those changes in terms of phenology and leaf chemistry could, in the end, affect ecosystem functioning; for instance, through litter decomposition processes. 

Since most of this work was based on secondary metabolism, further research needs to be conducted including other compounds, as it is well known that metabolites such as carbohydrates and amino acids can play important roles against drought stress. On the whole, data from this study will contribute to anticipating future climate-related changes in plant metabolomics and related ecosystem functioning although further research at longer time scales (i.e., 10 years of recurrent drought) are necessary, since Downy oak is one of the main drought-resistant tree species. 

## Figures and Tables

**Table 1:** Two-way PERMANOVA (999 permutations) performed on targeted and untargeted data to distinguish the effects of factors (season and drought) and their interactions (n = 5). F represents the dispersion of the data set and the significant *p*-value are indicated in bold.

	Targeted Analysis				Untargeted Analysis					
	2014		2015		2013		2014		2015	
Factors	*F*	*p*-Value	*F*	*p-*Value	*F*	*p-*Value	*F*	*p-*Value	*F*	*p-*Value
Season	*7.7*	**0.001**	*7.2*	**0.001**	*7.0*	**0.001**	*4.7*	**0.001**	*3.9*	**0.001**
Drought	*3.9*	**0.003**	*2.8*	**0.011**	*1.3*	0.168	*1.5*	0.091	*1.2*	0.191
Drought x Season	0.2	0.2	*0.8*	0.08	*0.9*	0.576	*0.8*	0.8	*1.0*	0.384

**Table 2:** Examples of downregulated drought biomarkers and their annotations, detected in tree leaves from AD plot, and highlighted through the construction of heatmaps and Venn diagrams. Annotations were performed by the comparison of experimental MS^2^ spectra with in silico fragmentation of metabolites from PubChem libraries using the MetFrag tool. Matches with raw formulae were also searched in the Metlin database (last accession: 6 October 2021). RT: retention time (s); TIC: Total Ion Chromatogram; CAS N°: Chemical Abstract Service Number. See the Supplementary Materials, “Supplementary excel file: metabolites annotation” for more biomarkers. + means an upregulation, whereas - means a downregulation with spring 2013, spring 2014, spring 2015|summer 2013, summer 2014, summer 2015|autumn 2013, autumn 2014, autumn 2015. _ means that no regulation was detected.

Feature	*m*/*z*	RT (s)	Max Intensity (Counts/% TIC)	Molecular Formula	Error (ppm)	mSigma ^†^	Seasons|Years of DownRegulation	Annotations
**Quercetin Derivatives**								
M619T573	617.1141	573	146,104-100.0	C_28_H_26_O_16_	1.1	2.5	_-_|--_|_--	dihydroquercetin galloyl hexoside
M477T578	477.0679	578	1,730,078-100.0	C_21_H_18_O_13_	1.3	10.7	___|---|--_	quercetin hexopyranosiduronic acid (glucuronide ?)
M595T604	595.1312	604	921,319-100.0	C_26_H_28_O_16_	−1.3	13.4	-__|---|---	quercetin apiosyl hexoside
M434T716_1	433.078	716	475,450-100.0	C_20_H_18_O_11_	−0.2	18.8	+__|_--|---	quercetin pentoside
M625T902	625.1196	902	125,813-100.0	C_30_H_26_O_15_	0.4	5.9	+_-|--_|_-_	quercetin caffeoyl hexoside
**Kaempferol Derivatives**								
M461T620	461.073	620	806,767-100.0	C_21_H_18_O_12_	−1.1	15	__-|__-|---	kaempferol hexopyranosiduronic acid (glucuronide ?)
M447T723	447.0926	723	587,915-100.0	C_21_H_20_O_11_	0.2	14.4	__-|_-_|-_-	kaempferol hexoside (astragalin ?)
M448T758	447.0927	758	1,765,218-100.0	C_21_H_20_O_11_	1.3	20.9	_+_|_+_|--_	kaempferol hexoside (astragalin ?)
M635T948	635.1392	948	411,290-100.0	C_32_H_28_O_14_	2.3	0.9	___|---|_--	kaempferol acetyl-*p*-coumaroyl hexoside
M782T953	781.1776	953	4,795,777-100.0	C_41_H_34_O_16_	−0.3	4.6	___|--_|---	kaempferol acetyl-di-*p*-coumaroyl hexoside
M823T957	823.1877	957	377,063-100.0	C_43_H_36_O_17_	0.3	1.6	-__|--_|---	kaempferol diacetyl-di-*p*-coumaroyl hexoside

^†^: mSigma is a constructor quality index that compares isotopic ratio and mass deviation of experimental ions relative to proposed theorical formulae. The lower the index, the more accurate the result.

**Table 3:** Examples of upregulated drought biomarkers and their annotation, detected in tree leaves from AD plot, and highlighted through the construction of heatmaps and Venn diagrams. Annotations were performed by the comparison of experimental MS^2^ spectra with in silico fragmentation of metabolites from PubChem libraries using the MetFrag tool. Matching with raw formulae were also searched in the Metlin database (last accession: 18 October 2021). RT: retention time (s); TIC: Total Ion Chromatogram; CAS N°: Chemical Abstract Service Number. See the Supplementary Materials, “Supplementary excel file: metabolites annotation” for more potential biomarkers. + means an upregulation whereas - means a downregulation with spring 2013, spring 2014, spring 2015|summer 2013, summer 2014, summer 2015|autumn 2013, autumn 2014, autumn 2015. _ means that no regulation was detected.

Feature	*m*/*z*	RT (s)	Max Intensity (Counts/% TIC)	Molecular Formula	Error (ppm)	mSigma ^†^	Seasons|Years of UpRegulation	Annotations	CAS Number (Possible Metabolites)
**Rhododendrin derivatives**									
M459T534	459.1869	573	146,104-100.0	C_21_H_32_O_11_	0.2	17.3	++_|++_|+_+	apiosyl rhododendrin	146609-83-8
M328T560	327.1447	560	37,232-76.6	C_16_H_24_O_7_	0.6	7.8	+_+|+++|+++	epirhododendrn	74390-35-5
M327T590	327.1437	590	37,021-44.5	C_16_H_24_O_7_	3.7	5.6	+++|_+_|+__	Rhododendrin ^†††^	497-78-9
M541T621	541.2273	621	1,029,013-100.0	C_26_H_38_O_12_	2.3	20.8	+++|_+_|+_+	-	
M480T720	479.1579	720	1,761,279-100.0	C_23_H_28_O_11_	−2.2	13.7	+++|_+_|++_	rhododendrin gallate ^††^	339079-19-5
**Kaempferol derivative**									
M417T860	417.0831	860	612,088-100.0	C_20_H_18_O_10_	−0.9	22.1	+++|+++|+++	kaempferol pentoside ^††^	5041-67-8/99882-10-7 (juglanin) 61117-16-6

^†^: mSigma is a constructor quality index that compares isotopic ratio and mass deviation of experimental ions relative to proposed theorical formulae. The lower the index, the more accurate the result. ^††^: metabolite found in the molecular network. ^†††^: biomarker confirmed by commercial standard co-injection. Last Metlin accession: 3 July 2019.

**Table 4:** Temperature (°C) and photosynthetically active radiation (PAR, µmol m^−2^ s^−1^) one week before (T_b_ and PAR_b_) and during the field campaigns (T_d_ and PAR_d_). Values shown correspond to daily mean ± S.E. (n = 5), averaged between 12:00 and 15:00 (local time).

Year	Field Campaigns	T_b_	PAR_b_	T_d_	PAR_d_
2014	Spring	12.7 ± 1.0	1189 ± 85	17.9 ± 0.6	1210 ± 107
	Summer	18.6 ± 0.5	1332 ± 255	26.2 ± 1.1	1351 ± 65
	Autumn	21.0 ± 0.3	873 ± 82	19.4 ± 1.0	676 ± 112
2015	Spring	25.1 ± 0.5	1377 ± 38	20.4 ± 0.9	1248 ± 85
	Summer	32.1 ± 0.2	1394 ± 99	29.5 ± 0.8	1379 ± 54
	Autumn	20.1 ± 0.6	578 ± 72	19.9 ± 0.6	1013 ± 44

## Data Availability

Data is contained within the article or supplementary material.

## Supplementary Materials

The following supporting information can be downloaded at: https://www.mdpi.com/article/10.3390/metabo12040307/s1. Table S1: Vapor-pressure deficit (VPD, kPa), carbon content (C, mg·g^−1^), nitrogen content (N, mg.g^−1^), and carbon:nitrogen ratio (C:N); Table S2: Pairwise comparisons performed after two-way PERMANOVA testing on metabolic profiles (targeted analysis); Table S3: Compound amounts (mg·g_DM_^−1^) according to drought treatments and seasons in 2014 and 2015; Table S4: Pairwise comparisons performed after two-way PERMANOVA on metabolic fingerprints (untargeted analyses); Figure S1: Molecular network obtained through Global Natural Products Social molecular networking and visualized with Cytoscape; Figure S2: Principal Component Analysis (PCA) on metabolic fingerprints according to drought treatment and seasons in 2013 (left), 2014 (middle), and 2015 (right), Figure S3: Mass spectra of rhododendrin (commercial standard) acquired in negative and positive modes with UHPLC-QToF at 10, 20, and 40 eV; Figure S4: Chromatogram and mass spectrum of drought biomarker annotated as rhododendrin (see Table 3 of the main manuscript) acquired at 20 eV in negative mode with UHPLC-qToF; Figure S5: Mass spectrum of drought biomarker annotated as apiosyl rhododendrin (see Table 3 of the main manuscript) acquired at 46.8 eV in negative mode with UHPLC-qToF; Supplementary excel file: metabolites annotation.
