# Supplementary material for: Amplified Drought and Seasonal Cycle Modulate Quercus pubescens Leaf Metabolome
Source: Metabolites. 2022 Mar 30;12(4):307. doi: 10.3390/metabo12040307 (PMC9026387; doi:10.3390/metabo12040307)
Supplement: Supplementary file 1 [file metabolites-12-00307-s001.zip › metabolites-1621104- supplementary materials.pdf]

Table S1. Vapor Pressure Deficit (VPD, kPa), carbon content (C, $mg.g^{-1}$), nitrogen content (N, $mg.g^{-1}$) and carbon:nitrogen ratio (C:N) according to seasons and drought treatments in 2014 and 2015. Differences between Natural Drought (ND) and Ampflified Drought (AD) were tested with Student tests at each season (mean ± S.E., n = 5). Letters denote differences between treatments for each season with a > b (highlighted in bold).

**2014**	**Spring**		**Summer**		**Autumn**	
	**ND**	**AD**	**ND**	**AD**	**ND**	**AD**
VPD	2.1 ± 0.1 ^{a}	1.9 ± 0.2 ^{a}	2.3 ± 0.3 ^{a}	2.4 ± 0.2 ^{a}	1.2 ± 0.1 ^{a}	1.4 ± 0.2 ^{a}
C	474.1 ± 3.7 ^{a}	463.6 ± 6.5 ^{a}	442.7 ± 13.2 ^{a}	460.0 ± 27.4 ^{a}	476.5 ± 7.8 ^{a}	487.4 ± 3.8 ^{a}
N	16.9 ± 1.1 ^{a}	16.5 ± 2.4 ^{a}	17.6 ± 3.3 ^{a}	18.5 ± 4.1 ^{a}	15.2 ± 2.3 ^{a}	15.8 ± 0.6 ^{a}
C:N	28.1 ± 0.6 ^{a}	28.5 ± 1.7 ^{a}	25.7 ± 1.7 ^{a}	25.4 ± 1.6 ^{a}	31.8 ± 1.7 ^{a}	30.9 ± 0.6 ^{a}
**2015**	**Spring**		**Summer**		**Autumn**	
	**ND**	**AD**	**ND**	**AD**	**ND**	**AD**
VPD	2.1 ±0.2 ^{a}	1.9 ± 0.3 ^{a}	3.0 ± 0.1 ^{a}	3.0 ± 0.1 ^{a}	1.4 ± 0.2 ^{a}	2.0 ± 0.3 ^{a}
C	460.0 ± 3.6 ^{a}	459.3 ± 7.7 ^{a}	476.7 ± 3.5 ^{a}	469.9 ± 2.9 ^{a}	472.5 ± 3.1 ^{a}	475.2 ± 14.9 ^{a}
N	18.3 ± 0.7 ^{a}	17.2 ± 1.4 ^{a}	16.9 ± 0.5 ^{a}	16.3 ± 0.6 ^{a}	**15.6 ± 0.2 ^{a}**	**14.3 ± 0.5 ^{b}**
C:N	25.3 ± 1.0 ^{a}	27.3 ± 2.3 ^{a}	28.3 ± 0.8 ^{a}	28.9 ± 1.2 ^{a}	30.4 ± 0.4 ^{a}	33.4 ± 1.3 ^{a}

Table S2. Pairwise comparisons performed after two-ways PERMANOVA test on metabolic profiles (targeted analysis) according to drought treatments and seasons in 2014 and 2015. Significant *P*-values ($p < 0.05$), were adjusted by False Discovery Rate approach. Relevant comparisons, season by season, relative to drought treatments are figured in grey (significant *P*-value in bold). ND = Natural Drought, AD = Amplified Drought.

**2014**	***Autumn AD***	***Autumn ND***	***Spring AD***	***Spring ND***	***Summer AD***
Autumn ND	0.10				
Spring AD	0.04	0.05			
Spring ND	0.04	0.05	0.10		
Summer AD	0.05	0.10	0.03	0.06	
Summer ND	0.05	0.05	0.03	0.04	0.60

**2015**	***Autumn AD***	***Autumn ND***	***Spring AD***	***Spring ND***	***Summer AD***
Autumn ND	0.41				
Spring AD	0.03	0.04			
Spring ND	0.03	0.04	**0.04**		
Summer AD	0.03	0.10	0.03	0.04	
Summer ND	0.03	0.04	0.03	0.03	0.10

Table S3. Compound amounts (mg.g_{DM}^{-1}) according to drought treatments and seasons in 2014 and 2015. Mean ± S.E. (n = 5). Student tests were performed for each season to test significant differences ($P < 0.05$, highlighted in bold) in phenolic concentrations between amplified (AD) and natural drought (ND). Letters denote significant differences between groups with a > b. RT = retention time (min), Theor. ion mass = theorical ion mass (mass unit), Exp. ion mass = experimental ion mass (mass unit).

						Spring		Summer		Autumn	
**2014**	**Chemical groups**	**Compounds**	**RT (min)**	**Theor. ion mass**	**Exp. ion mass**	**ND**	**AD**	**ND**	**AD**	**ND**	**AD**
Flavonoids	Flavonols	myricetin hexose	8.23	479	479	0.6 ± 0.2	0.2 ± 0.1	0.9 ± 0.5	0.7 ± 0.4	0.01 ± 0.01	0.0 ± 0.0
		quercetin pentose hexose	8.64	595	595	0.08 ± 0.04	0.04 ±0.02	**0.2 ± 0.1** [a]	**0.01±0.01** [b]	0.13 ± 0.04	0.05 ± 0.02
		quercetin galloyl glucose	9.14	615	615	0.6 ± 0.1	0.3 ± 0.03	1.1 ± 0.3	0.8 ± 0.1	**1.1 ± 0.2** [a]	**0.6 ± 0.1** [b]
		myricitrin	9.64	463	463	**1.9 ± 0.3** [a]	**1.3 ± 0.1** [b]	**2.3 ± 0.7** [a]	**1.7 ± 0.2** [b]	1.7 ± 0.3	1.1± 0.1
		quercetin-3-*O*-glucose	9.89	463	463	**3.9 ± 0.5** [a]	**2.5 ± 0.3** [b]	3.2 ± 0.5	3.1 ± 0.6	2.5 ± 0.3	2.2 ± 0.3
		quercetin hexose 1	9.80	-	477	1.2 ± 0.1	0.9 ± 0.2	1.0 ± 0.2	0.9 ± 0.3	0.7 ± 0.1	0.5 ± 0.1
		flavonoid galloyl hexose	10.69	599	599	0.3 ± 0.03	0.2 ± 0.1	0.3 ± 0.1	0.3 ± 0.1	0.3 ± 0.03	0.3 ± 0.04
		kaempferol hexose	11.03	447	447	3.1 ± 0.5	2.7 ± 0.3	1.5 ± 0.1	1.9 ± 0.4	1.3 ± 0.2	1.5 ± 0.2
		kaempferol-3-glucose	11.15	447	447	0.1 ± 0.1	0.1 ± 0.1	0.3 ± 0.1	0.2 ± 0.1	0.5 ± 0.1	0.5 ± 0.2
		quercetin hexose 2	11.32	-	477	1.4 ± 0.3	1.9 ± 0.5	1.5 ± 0.4	2.2 ± 0.7	1.4 ± 0.4	1.5 ± 0.4
		isorhamnetin-3-glucuronide	11.45	491	491	0.6 ± 0.2	0.6 ± 0.1	0.6 ± 0.2	0.6 ± 0.1	0.5 ± 0.2	0.41 ± 0.03
	Flavanols	catechin	5.01	289	289	0.8 ± 0.4	0.6 ± 0.1	1.2 ± 0.3	1.3 ± 0.3	1.7 ± 0.6	1.5 ± 0.2
		epigallocatechin caffeate	5.70	467	467	4.4 ± 0.8	2.7 ± 0.4	2.7 ± 0.7	1.8 ±0.4	2.7 ± 0.7	1.6 ± 0.4
Phenolic acids		*p*-coumaroyl quinic acid	4.67	337	337	**3.2 ± 0.3** [a]	**2.1 ± 0.3** [b]	1.2 ± 0.1	1.0 ± 0.2	0.7 ± 0.1	0.5 ± 0.1
		feruloyl quinic acid	5.41	367	367	0.8 ± 0.1	0.6 ± 0.1	0.32 ± 0.03	0.2 ± 0.02	**0.22± 0.02** [a]	**0.15±0.01** [b]
		gallic acid conjugate	2.76	466	-	4.6 ± 0.4	4.0 ± 0.6	1.6 ± 0.1	1.8 ± 0.2	0.6 ± 0.1	0.6 ± 0.2
**2015**											
Flavonoids	Flavonols	myricetin hexose	8.23	479	479	0.03 ± 0.01	0.03 ±0.01	0.09 ± 0.03	0.02 ± 0.02	0.0 ± 0.0	0.03 ± 0.03
		quercetin pentose hexose	8.64	595	595	0.08 ± 0.04	0.03 ±0.01	0.2 ± 0.1	0.1 ± 0.02	0.1 ± 0.03	0.03 ± 0.02
		quercetin galloyl glucose	9.14	615	615	0.6 ± 0.2	0.3 ± 0.02	0.9 ± 0.2	0.5 ± 0.04	0.6 ± 0.2	0.7 ± 0.03
		myricitrin	9.64	463	463	**1.2 ± 0.9** [a]	**0.7 ± 0.1** [b]	**1.1 ± 0.1** [a]	**0.7 ± 0.1** [b]	1.1 ± 0.3	0.9 ± 0.1
		quercetin-3-glucose	9.89	463	463	2.4 ± 0.5	1.5 ± 0.1	2.0 ± 0.2	1.5 ± 0.2	2.2 ± 0.2	2.0 ± 0.2
		quercetin hexose 1	9.80	-	477	**0.9 ± 0.1** [a]	**0.6 ± 0.1** [b]	0.5 ± 0.03	0.4 ± 0.1	0.7 ± 0.1	0.5 ± 0.1

		flavonoid galloyl hexose	10.69	599	599	0.3 ± 0.03	0.2 ± 0.1	0.3 ± 0.02	0.3 ± 0.04	0.3 ± 0.1	0.3 ± 0.04
		kaempferol hexose	11.03	447	447	1.9 ± 0.3	1.9 ± 0.1	**0.8 ± 0.1** [b]	**1.2 ± 0.1** [a]	1.3 ± 0.1	1.5 ± 0.1
		kaempferol-3-glucose	11.15	447	447	0.0 ± 0.0	0.0 ± 0.0	0.3 ± 0.1	0.5 ± 0.1	0.7 ± 0.1	0.7 ± 0.2
		quercetin hexose 2	11.32	477	-	1.1 ± 0.3	1.3 ± 0.3	1.1 ± 0.3	1.3 ± 0.4	1.2 ± 0.3	1.5 ± 0.3
		isorhamnetin-3-glucuronide	11.45	491	491	0.5 ± 0.1	0.4 ± 0.04	0.3 ± 0.1	0.3 ± 0.4	0.5 ± 0.1	0.5 ± 0.04
	Flavanols	catechin	5.01	289	289	1.0 ± 0.4	1.0 ± 0.1	1.2 ± 0.4	1.0 ± 0.03	1.9 ± 0.8	1.3 ± 0.1
		epigallocatechin caffeate	5.70	467	467	4.0 ± 0.8	3.4 ± 0.4	2.4 ± 0.6	1.8 ± 0.4	1.9 ± 0.7	1.5 ± 0.2
Phenolic acids		*p*-coumaroyl quinic acid	4.67	337	337	1.8 ± 0.4	2.6 ± 0.3	0.9 ± 0.1	0.6 ± 0.1	0.5 ± 0.1	0.4 ± 0.1
		feruloyl quinic acid	5.41	367	367	0.7 ± 0.1	0.5 ± 0.1	**0.28±0.02** [a]	**0.20 ± 0.02** [b]	**0.20 ± 0.02**[a]	**0.15± 0.01**[b]
		gallic acid conjugate	2.76	466	466	3.7 ± 0.4	4.6 ± 0.3	0.8 ± 0.1	1.1 ± 0.2	0.1 ± 0.04	0.2 ± 0.1

Table S4. Pairwise comparisons performed after two-ways PERMANOVA on metabolic fingerprints (untargeted analyses) according to drought treatments and seasons in 2013, 2014 and 2015. Significant *P*-values ($P < 0.05$) were adjusted by False Discovery Rate approach. Relevant comparisons, season by season, relative to drought treatments are figured in grey. ND = Natural Drought, AD = Amplified Drought.

**2013**	***Autumn ND***	***Autumn AD***	***SpringND***	***SpringAD***	***SummerND***
Autumn AD	0.62				
SpringND	0.04	0.04			
Spring AD	0.05	0.04	0.53		
SummerND	0.03	0.04	0.03	0.04	
SummerAD	0.03	0.05	0.04	0.04	0.24

**2014**	***Autumn ND***	***Autumn AD***	***SpringND***	***SpringAD***	***SummerND***
Autumn AD	0.30				
SpringND	0.02	0.02			
SpringAD	0.02	0.04	0.49		
SummerND	0.02	0.02	0.02	0.02	
SummerAD	0.02	0.02	0.02	0.02	0.24

**2015**	***Autumn ND***	***Autumn AD***	***SpringND***	***SpringAD***	***SummerND***
Autumn AD	0.63				
SpringND	0.04	0.04			
SpringAD	0.04	0.04	0.45		
SummerND	0.45	0.40	0.05	0.05	
SummerAD	0.42	0.45	0.04	0.07	0.42

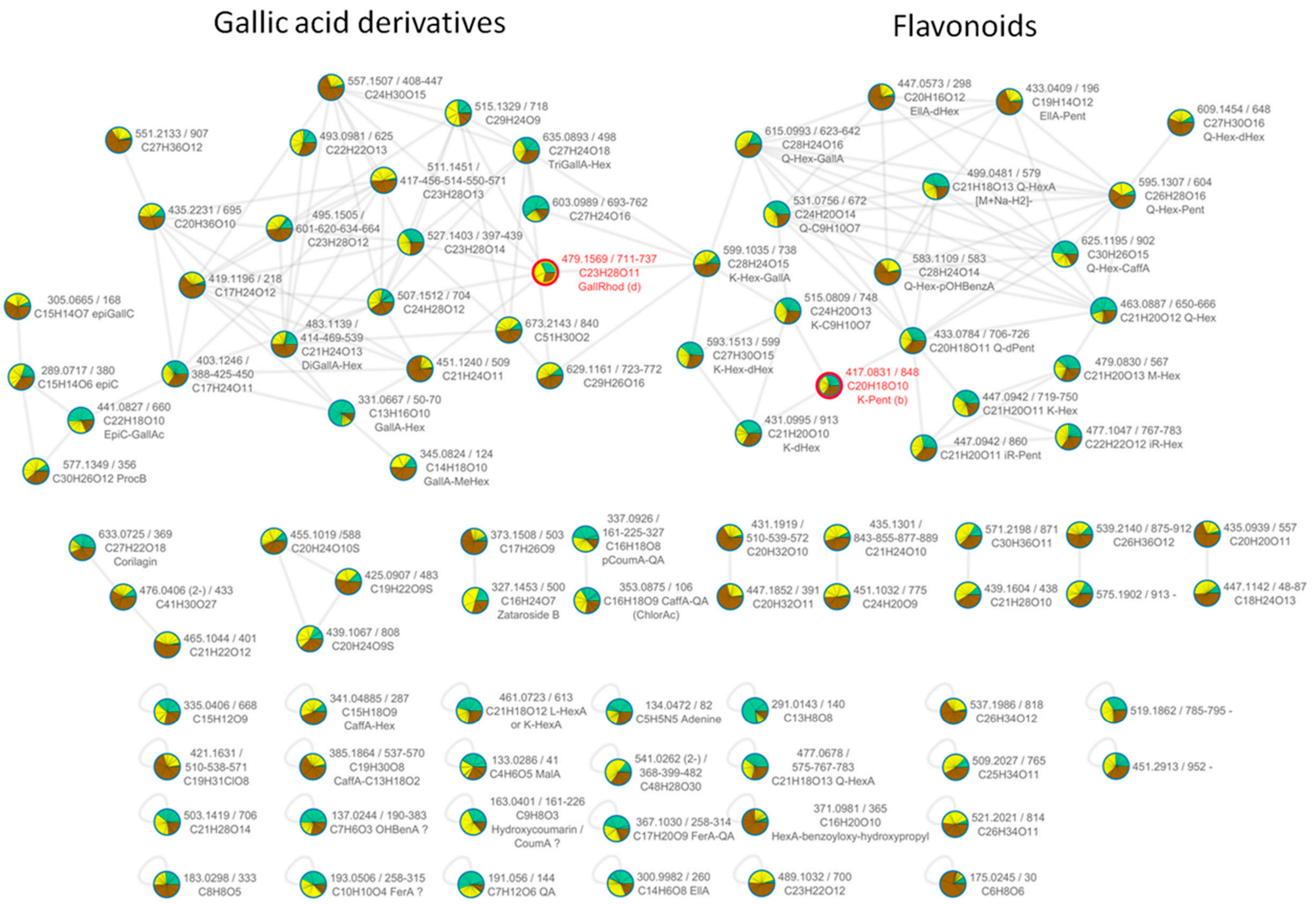

Figure S1. Molecular network obtained through Global Natural Products Social molecular networking and visualized with Cytoscape. Network is based on MS^2 spectra of major detected compounds (merged data from 2013 and 2014 of untargeted analysis) according to

seasons (green for spring, yellow for summer and brown for autumn). Pie charts are based on MS^1 quantitative data of the major metabolite since nodes can group isomers presenting fragmentation similarities at different retention times. On each node: ion exact mass (atomic mass unit) /retention times of isobars (seconds), molecular formula, annotation. Q: quercetin, K: kaempferol, Ir: isorhamnetin, M: myricetin, L: luteolin, ProcB: procyanidin B, Hex: hexose, HexA: Hexuronic acid, dHex: deoxyhexose, Pent: pentose, dPent: deoxypentose, MeHex: methyl hexose, EpiC: epicatechin, epiGallC: epigallocatechin, GallA: gallic acid, diGallA: digallic acid, triGallicA: trigallic acid, CaffA: caffeic acid, FerA: ferulic acid, EllA: ellagic acid, MalA: malic acid, QA: quinic acid, pCoumA: p-coumaric acid, GallRhod: galloylrhododendrin and pOHBenzA: p-hydroxybenzoic acid.

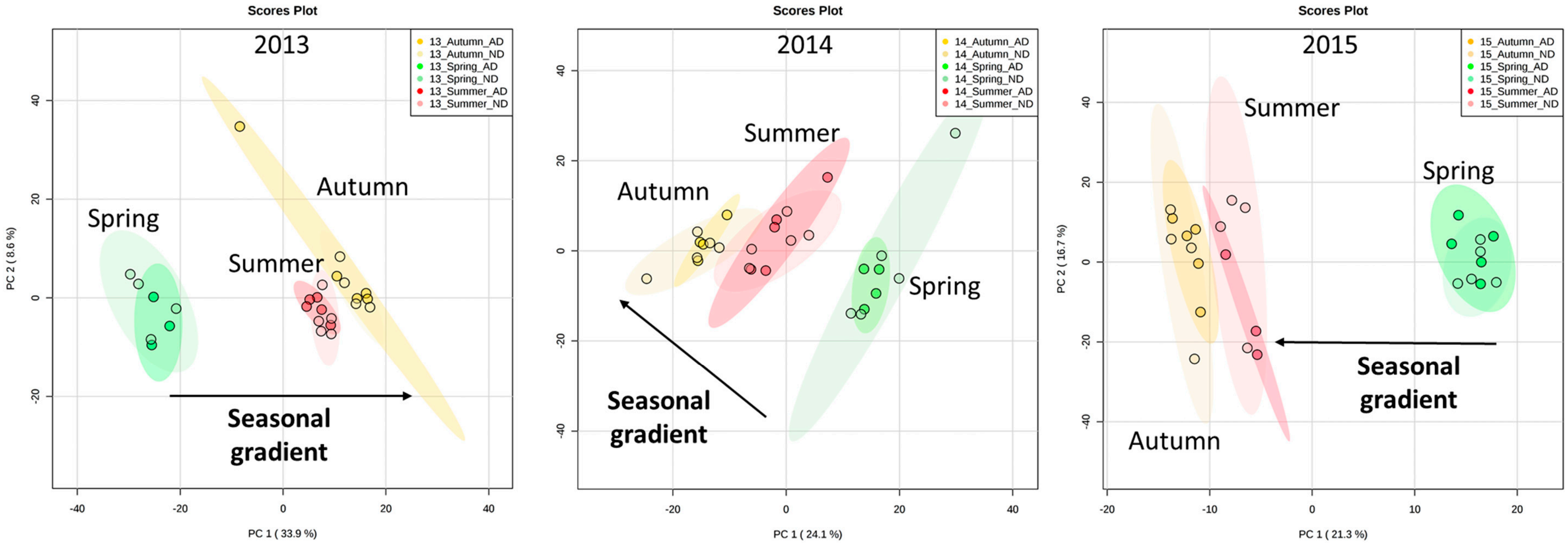

Figure S2. Principal Component Analysis (PCA) on metabolic fingerprints according to drought treatment and seasons in 2013 (left), 2014 (middle) and 2015 (right).

Rhododendrin (commercial standard) – $C_{16}H_{24}O_7$ – 328.1522 – RT 605 s

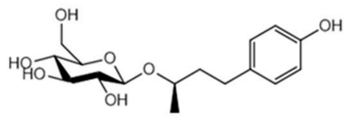

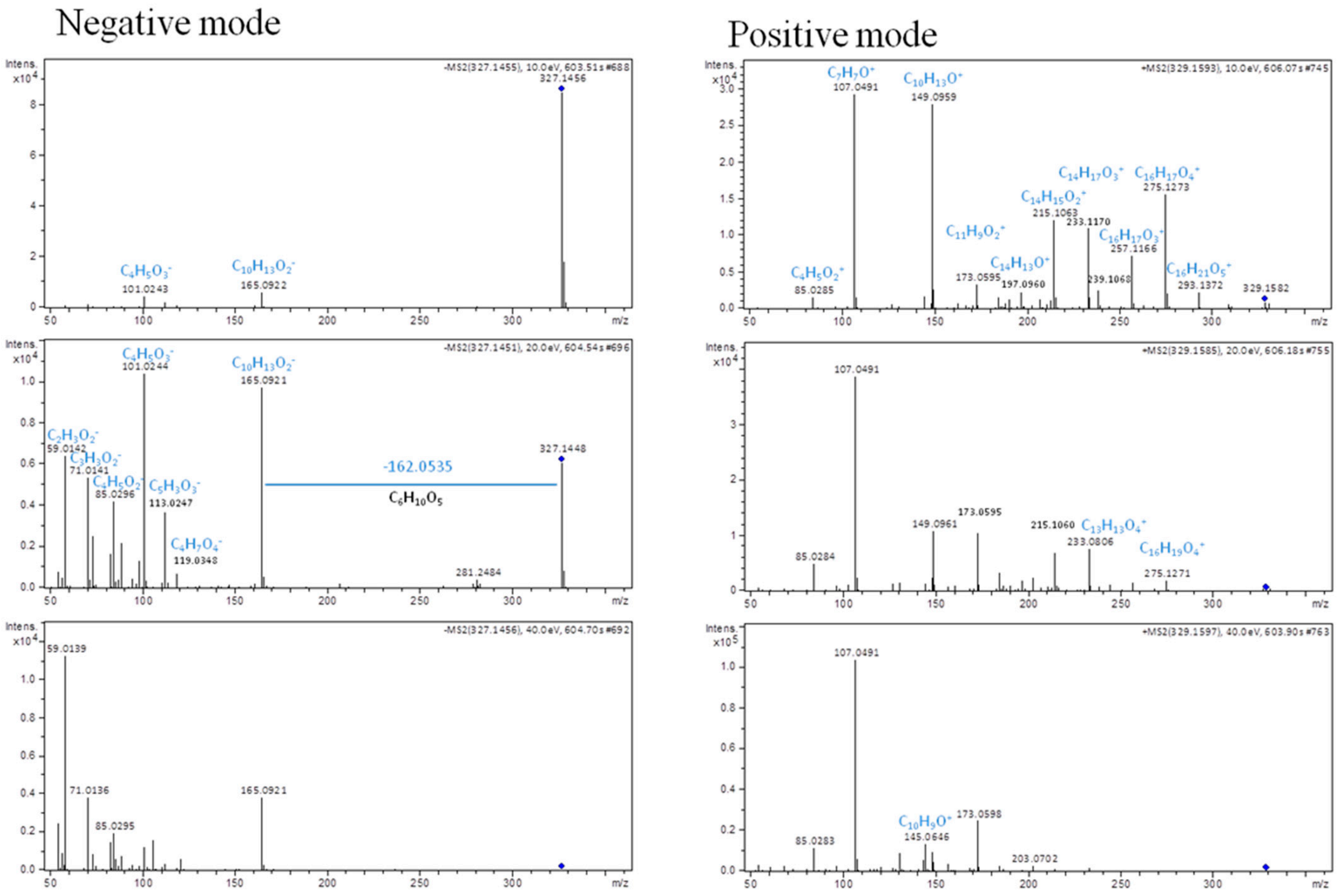

Figure S3. Mass spectra of rhododendrin (commercial standard) acquired in negative and positive modes with UHPLC-QToF at 10, 20 and 40eV.

a – *m/z* 327.1446 – RT 590-600 s
Annotated as rhododendrin

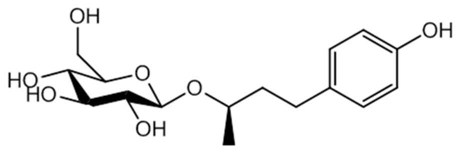

Negative mode

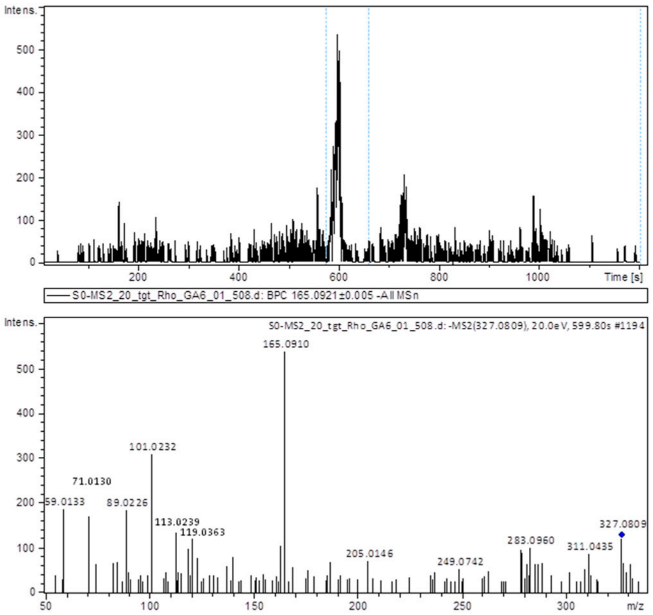

Positive mode

No experimental data

Figure S4. Chromatogram and mass spectrum of drought biomarker annotated as rhododendrin (see Table 3 of main manuscript) acquired at 20eV in negative mode with UHPLC-qToF.

c – *m/z* 459.1872 – RT 534 s
Annotated as apiosyl rhododendrin

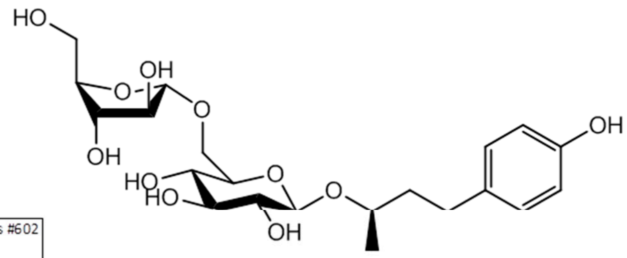

Experimental spectrum (negative mode)

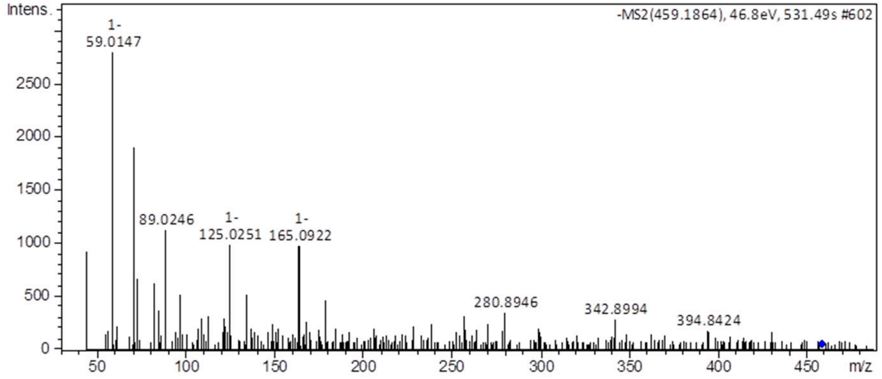

(-) 20eV

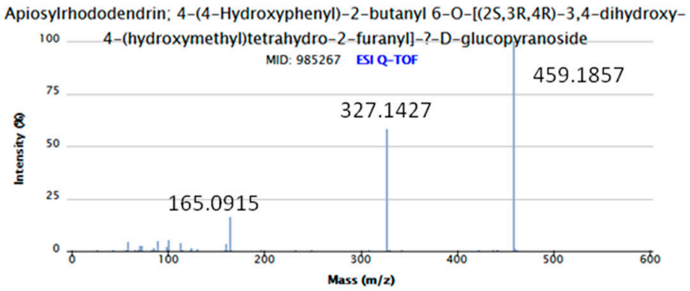

(-) 40eV

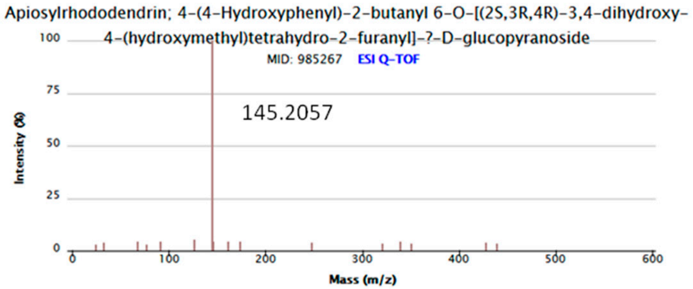

Figure S5. Mass spectrum of drought biomarker annotated as apiosyl rhododendrin (see Table 3 of main manuscript) acquired at 46.8 eV in negative mode with UHPLC-qToF.
